# Exposure of Human Gastric Cells to Oxidized Lipids Stimulates Pathways of Amino Acid Biosynthesis on a Genomic and Metabolomic Level

**DOI:** 10.3390/molecules24224111

**Published:** 2019-11-14

**Authors:** Mathias Zaunschirm, Marc Pignitter, Antonio Kopic, Claudia Keßler, Christina Hochkogler, Nicole Kretschy, Mark Manuel Somoza, Veronika Somoza

**Affiliations:** 1 Department of Physiological Chemistry, Faculty of Chemistry, University of Vienna, 1090 Vienna, Austria; 2 Department of Inorganic Chemistry, Faculty of Chemistry, University of Vienna, 1090 Vienna, Austria

**Keywords:** linoleic acid peroxidation products, hexane, gastric cells, metabolomics, cDNA microarray

## Abstract

The Western diet is characterized by a high consumption of heat-treated fats and oils. During deep-frying processes, vegetable oils are subjected to high temperatures which result in the formation of lipid peroxidation products. Dietary intake of oxidized vegetable oils has been associated with various biological effects, whereas knowledge about the effects of structurally-characterized lipid peroxidation products and their possible absorption into the body is scarce. This study investigates the impact of linoleic acid, one of the most abundant polyunsaturated fatty acids in vegetable oils, and its primary and secondary peroxidation products, 13-HpODE and hexanal, on genomic and metabolomic pathways in human gastric cells (HGT-1) in culture. The genomic and metabolomic approach was preceded by an up-to-six-hour exposure study applying 100 µM of each test compound to the apical compartment in order to quantitate the compounds’ recovery at the basolateral side. Exposure of HGT-1 cells to either 100 µM linoleic acid or 100 µM 13-HpODE resulted in the formation of approximately 1 µM of the corresponding hydroxy fatty acid, 13-HODE, in the basolateral compartment, whereas a mean concentration of 0.20 ± 0.13 µM hexanal was quantitated after an equivalent application of 100 µM hexanal. An integrated genomic and metabolomic pathway analysis revealed an impact of the linoleic acid peroxidation products, 13-HpODE and hexanal, primarily on pathways related to amino acid biosynthesis (*p* < 0.05), indicating that peroxidation of linoleic acid plays an important role in the regulation of intracellular amino acid biosynthesis.

## 1. Introduction

In industrialized countries, the habitual diet is characterized by a high intake in dietary fats, mainly originating in heat-treated foods [1]. Among them, the popularity of deep-fried products is based on convenience, their crispy texture, and pleasant mouth feel compared to non-fried foods [2]. During the deep-frying process, the frying oils are severely heated, resulting in multiple chemical reactions of the oils’ constituents [3]. One of the major determinants of the quality of a frying oil are lipid peroxidation products. Here, primary and secondary lipid peroxidation products are formed through autoxidation or photo-oxidation reactions of unsaturated fatty acids, resulting in the generation of lipid hydroperoxides and further decomposition products, such as aldehydes, carboxylic acids, alcohols, or hydrocarbons [4]. Under thermal treatment, free fatty acids undergo faster oxidation processes than under non-heated conditions [5]. The free fatty acid, linoleic acid, is an essential unsaturated fatty acid and the predominant omega-6 polyunsaturated fatty acid in the Western diet. Linoleic acid can be found in vegetable oils commonly used for deep-frying, such as rice bran, safflower, sunflower, soybean, corn, and canola oil [6,7]. Other prominent sources of linoleic acid, and therefore prone to oxidation, are walnuts, pine nuts, and pecans [8].

In several animal feeding studies, highly oxidized fats with peroxide values >10 meq/kg oil have been administered [9,10,11,12]. On the one hand, oxidized fats and oils have been hypothesized to be harmful to health and associated with, e.g., the development of atherosclerosis [13]. On the other hand, animal feeding studies have also shown that dietary oxidized fats lead to a decrease in triacylglycerols and cholesterol in liver and plasma, and to the regulation of genes involved in lipid metabolism [14]. However, studies on the effects of structurally-characterized primary and secondary peroxidation products of linoleic acid are lacking. Moreover, it has been shown that the primary peroxidation product of linoleic acid, 13-hydroperoxy-9*Z*,11*E*-octadecadienoic acid (13-HpODE), is mainly decomposed in the stomach into secondary peroxidation products such as its corresponding alcohols, epoxyketones and aldehydes, which were demonstrated to be partially incorporated into the intestinal lumen and further absorbed by enterocytes [10,15].

Since the stomach is a very reactive environment for the chemically rather instable dietary lipid peroxidation products [16], the presented work addresses whether linoleic acid and its primary and secondary peroxidation products, 13-HpODE and hexanal, are absorbed by human gastric cells (HGT-1) and show pre-absorptive cellular effects on genomic and metabolic levels.

## 2. Results

### 2.1. Quantitative Recovery of Linoleic Acid and Its Peroxidation Products in the Basolateral Compartment of HGT-1 Cells after Apical Exposure 

After six hours of incubation with 100 µM linoleic acid, 13-HODE but no linoleic acid could be quantitated in the apical and basolateral compartment of the HGT-1 cells, respectively (Table 1). Treatment with 100 µM 13-HpODE revealed a 71% higher concentration of 13-HODE in the basolateral compartment of the cells compared to the apical compartment (*p* ≤ 0.05), whereas the concentration of 13-HpODE did not reach the limit of detection (LOD: 0.1 µg/mL). Moreover, a substantially higher amount of 13-HODE (+ 87%) was quantitated in the cells’ basolateral compartment after exposure to 13-HpODE compared to the treatment with linoleic acid (*p* ≤ 0.05). After a 30 min incubation period with 100 µM hexanal, hexanal could be quantitated in the apical as well as basolateral compartments. Neither 13-HODE nor hexanal were detected in the lysates of HGT-1 cells.

### 2.2. Genomic Analysis of RNA

**Figure 1:**
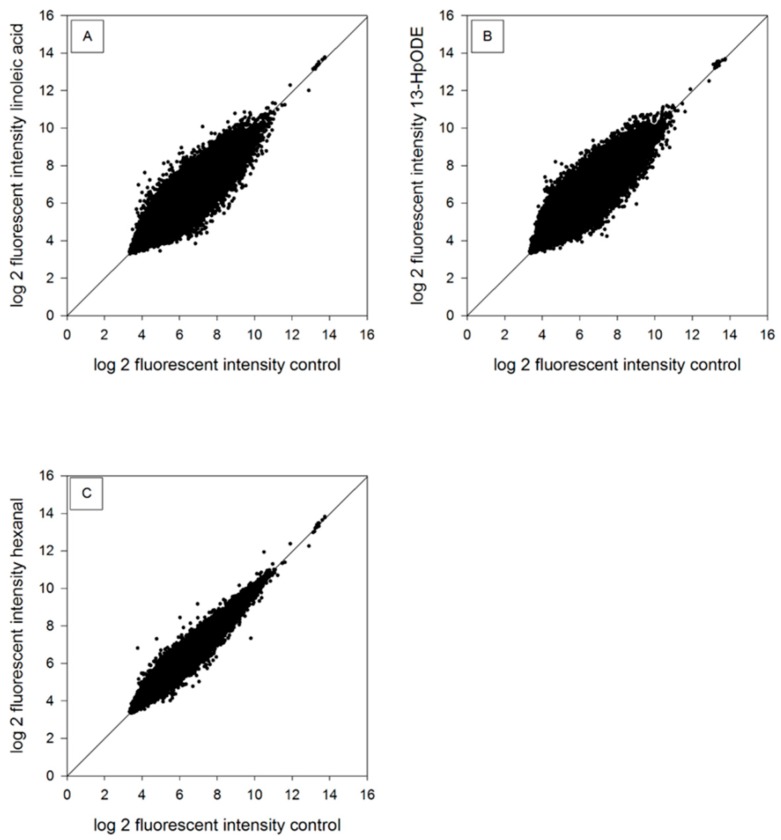
Scatterplots of log2 fluorescence intensity after six-hours incubation with (**A**) 100 µM linoleic acid, (**B**) 100 µM 13-HpODE or (**C**) 100 µM hexanal in HGT-1 cells. Diagonal represents equal regulation in untreated control and treated samples (*n* = 3).

The impact of linoleic acid, 13-HpODE, and hexanal at the genomic level of HGT-1 cells was analyzed using a customized cDNA microarray [17]. The scatterplots show log base 2 fluorescence intensity after a six-hour incubation period with 100 µM linoleic acid, 100 µM 13-HpODE or 100 µM hexanal in HGT-1 cells (Figure 1). Treatment with 100 µM linoleic acid resulted in 1303 regulated probes (2.78% of all probes). Exposure of the cells to 100 µM 13-HpODE revealed 420 regulated probes (0.90% of all probes), whereas cells treated with 100 µM hexanal showed 193 regulated probes (0.41% of all probes) compared to medium-only treated cells. The standard threshold for regulation, either a ≤0.8 or ≥1.2-fold change benchmark for an altered gene expression, was used [18]. The software DAVID (database for annotation, visualization and integrated discovery) was used for analysis and to generate individual functional annotation clusters for gene expression changes of genes with similar biological properties and to show different enrichment scores (ES). Resulting annotation clusters with an enrichment score ≥1.3 are considered to be of statistical relevance [19]. After six hours of incubation with 100 µM linoleic acid, 100 µM 13-HpODE or 100 µM hexanal, selected annotation clusters with enrichment scores ≥1.3 were found (supplementary data, Table S1) to be involved in gene regulation of “tRNA splicing endonuclease subunit 2 (TSEN2)” (in annotation cluster: “mRNA processing”, ES: 1.33)), “general transcription factor IIH subunit 1 (GTF2H1)” (in annotation cluster: “transcription initiation from RNA polymerase II promoter processing”, ES: 1.62)) and “trace amine associated receptor 1 (TAAR1)” (in annotation cluster: “topological domain: Cytoplasmic”, ES: 3.28)), respectively. 

### 2.3. Metabolomic Analysis

**Figure 2:**
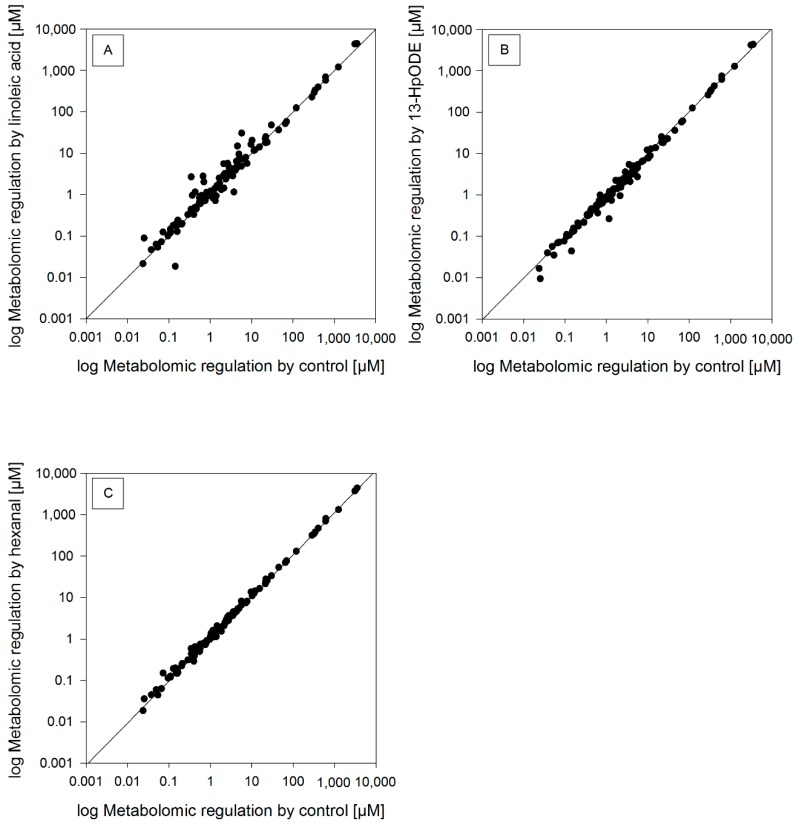
Scatterplots of metabolomic regulation [log µM] after six-hours incubation with (**A**) 100 µM linoleic acid, (**B**) 100 µM 13-HpODE, and (**C**) 100 µM hexanal in HGT-1 cells. Bisector represents equal regulation in untreated control and treated samples (*n* = 3–4).

The impact of linoleic acid, 13-HpODE, and hexanal at the metabolic level of HGT-1 cells was examined by applying a pathway analysis at the metabolomic level. The scatterplots (Figure 2) show the metabolomic regulation [µM] after a six-hour incubation with 100 µM linoleic acid, 100 µM 13-HpODE, or 100 µM hexanal in HGT-1 cells. Treatment with 100 µM linoleic acid resulted in 49 regulated probes (30.1% of all probes), with 100µM 13-HpODE in 40 regulated probes (24.5% of all probes) and with 100 µM hexanal in 32 regulated probes (19.6% of all probes), which showed either ≤0.8 or ≥1.2-fold change used as a benchmark for potential changed metabolites [20]. With these probes, a metabolic pathway analysis was performed using MetaboAnalyst 3.6, revealing different pathways affected by changed metabolites, showing different *p*-values (*p*-values less than 0.05 show statistical relevance). Metabolic pathway analysis revealed that treatment with 100 µM linoleic acid altered metabolites affecting “Glycerophospholipid metabolism” (*p* = 0.007), “Aminoacyl-tRNA biosynthesis” (*p* = 0.020) and “Linoleic acid metabolism” (*p* = 0.049). Top pathways influenced after incubation with 10 0 µM 13-HpODE were shown to be “Aminoacyl-tRNA biosynthesis” (*p* < 0.001) and “Glycerophospholipid metabolism” (*p* = 0.011). An incubation with 100 µM hexanal resulted in a major impact of the metabolic pathways “Aminoacyl-tRNA biosynthesis” (*p* < 0.001), “D-Arginine and D-Ornithine metabolism” (*p* < 0.001) and “Valine, Leucine, Isoleucine“ (*p* = 0.008) (supplementary data, Table S2). These results indicate an alteration of endogenous metabolites affecting especially the metabolic pathway of the aminoacyl-tRNA biosynthesis after a six-hour incubation period.

### 2.4. Combined Genomic and Metabolomic Effects of Linoleic Acid and Its Lipid Peroxidation Products on HGT-1 Cells: An Integrated Pathway Analysis

**Figure 3:**
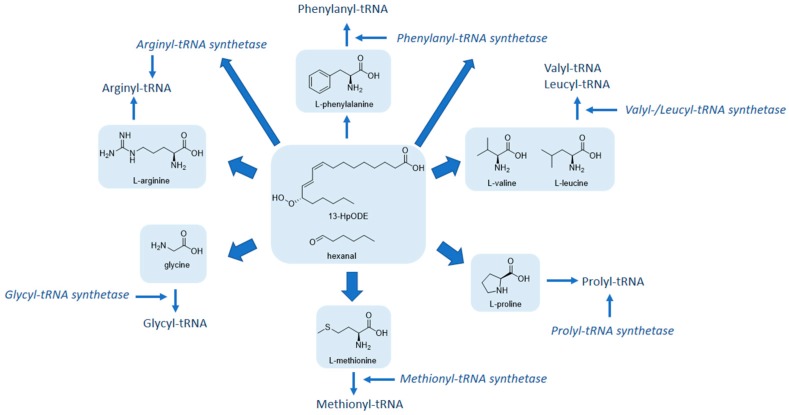
Integrated pathway analysis of genomic and metabolomic data revealed pathways related to amino acid biosynthesis being influenced by 13-HpODE and hexanal in gastric cells as indicated by thick arrows.

Since a regulation of gene expression does not necessarily result in functional changes of metabolic pathways, an integrated pathway analysis of the combined genomic and metabolomic results was performed by means of the freely available MetaboAnalyst 3.6 software, a comprehensive tool for metabolomics analysis and interpretation [20]. After incubation with linoleic acid, 13-HpODE, or hexanal, genes and metabolites showing a fold change of either ≤0.8 or ≥1.2 were uploaded and an integrated pathway analysis was carried out. The data was mapped to KEGG (Kyoto encyclopedia of genes and genomes) metabolic pathways and enrichment and topology analyses were performed. Enrichment analysis evaluates the appearance of affected genes or metabolites in specific pathways. Topology analysis estimates the biological importance of affected genes or metabolites based on their position within a pathway. Table 2 shows selected enriched genes and metabolites, with a *p*-value less than 0.05, being involved in certain pathways after six hours of incubation with 100 µM 13-HpODE or 100 µM hexanal in HGT-1 cells. The top-ranked pathways being influenced by enriched genes and metabolites in combination after incubation with 100 µM 13-HpODE were “Aminoacyl-tRNA biosynthesis” (*p* < 0.001, 6 hits out of 87), “Linoleic acid metabolism” (*p* = 0.03, 3 hits out of 34) and “Arginine and proline metabolism” (*p* < 0.05, 5 hits out of 102). Incubation with 100 µM 13-HpODE increased levels of the amino acids glycine, methionine, proline leucine/isoleucine by 1.30, 1.26, 1.21, 1.28-fold, respectively, compared to non-treated controls (= 1). Incubation with 100 µM hexanal led to an enrichment of the pathways “Valine, leucine and isoleucine biosynthesis” (*p* < 0.001, 3 hits out of 13) and “Aminoacyl-tRNA biosynthesis” (*p* < 0.001, 5 hits out of 87). Moreover, treatment with 100 µM hexanal led to an upregulation of cellular levels of arginine (1.34-fold), glycine (1.20-fold), methionine (1.36-fold), valine (1.31-fold), leucine/isoleucine (1.33-fold) compared to untreated control cells (= 1). However, incubation with 100 µM linoleic acid did not result in a significant enrichment of any amino acid pathway. These outcomes indicate that predominantly pathways of the amino acid and protein biosynthesis were affected by the primary and secondary linoleic acid peroxidation products, 13-HpODE and hexanal, respectively, but not by the unoxidized linoleic acid (Figure 3).

## 3. Discussion

Consumption of thermally-treated and/or stored vegetable oils is accompanied by an intake of lipid peroxidation products generated during these processes [3,21]. Dietary intake of food-derived lipid peroxides is hypothesized to have adverse effects on human health, whereas studies on the effects of structurally-characterized lipid peroxidation products are lacking. There is evidence that ingested lipid peroxides undergo further reactions in the stomach during digestion, either forming further peroxidation products, or being decomposed, or partially incorporated by the gastric tissue [10,15,16]. Since linoleic acid is one of the quantitatively dominating fatty acids of vegetable oils, the exposure of human gastric cells in culture to linoleic acid and its primary and secondary peroxidation products, 13-HpODE and hexanal, was tested after six (linoleic acid and 13-HpODE) or 0.5 h (hexanal) of incubation, thereby simulating the range of retention times of fat during the gastric digestion in humans [22,23,24]. It has been shown in animal studies that the main degradation processes of linoleic acid hydroperoxides occur in the gastric environment [10,15]. Moreover, it has been demonstrated that 13-HpODE is not absorbed by intact Caco-2 intestinal cells. Instead they are likely to be metabolized by gastrointestinal glutathione peroxidase, which has been shown to be present in rats [25]. Therefore, the exposure study presented here was conducted in HGT-1 cells, based on the hypothesis that this cell model is more relevant than intestinal cells for investigating absorption mechanisms of linoleic acid and its peroxidation products. After six hours of incubation with 100 µM linoleic acid or 100 µM 13-HpODE, neither linoleic acid nor 13-HpODE were detectable in the apical compartment of HGT-1 cells, whereas 13-HODE could be quantitated. Also, HGT-1 cell exposure to 100 µM hexanal resulted in a mean detectable concentration of 0.20 ± 0.13 µM in the basolateral compartment, indicating the majority of hexanal might have been decomposed or evaporated after 0.5 h of incubation under the experimental conditions. It has to be noted that the degree of evaporation and/or decomposition was not investigated in the current study. It might also be conceivable that a lack of diffusion of hexanal in the system might be an explanation for the limited amount of hexanal detected on the basolateral side. Since linoleic acid and its peroxidation products are highly reactive compounds, their degradation during the six hours of incubation is conceivable, likely resulting in a conversion of linoleic acid and 13-HpODE to 13-HODE and many other decomposition products, which will need to be identified in future studies. Moreover, we cannot exclude metabolic or chemical modifications of the test compounds during the exposure study, e.g., formation of other, yet not identified peroxidation products or non-/covalent binding to proteins. An earlier study by Schieberle and Grosch [26] could show that, after three hours incubation at 38 °C in the presence of oxygen and di-tert-butyl peroxyoxalate, the incubated methyl 13-hydroperoxy-cis-9-trans-11-octadecadienoate was completely decomposed into a mixture consisting of oxo-, oxo-/epoxy-, hydroxy-, hydroxy-/epoxy-, oxo-/dihydroxy- compounds in a cell-free system. In our study, we demonstrated that 13-HODE and hexanal, both degradation products of 13-HpODE, were quantitated at the basolateral side of HGT-1 cells cultured in a transwell system and exposed to linoleic acid, 13-HpODE, or hexanal. This experiment revealed an 87% higher amount of 13-HODE at the basolateral side of the cells after incubation with 13-HpODE than with linoleic acid at the equimolar concentrations applied. An explanation for this result could be that 13-HpODE is a direct precursor of 13-HODE [5]. Linoleic acid, in contrast, would need to be first oxidized into 13-HpODE before its possible cleavage into 13-HODE, making 13-HpODE a better source of 13-HODE [5]. The incorporation of linoleic acid hydroxide into gastric tissue was also shown after intragastric administration of linoleic acid hydroperoxides to rats. Moreover, an application of linoleic acid hydroxide as single compound led to an 80% decrease during four hours in the gastric environment [10]. Ramsden et al. [27] could show that after a twelve-week intervention study lowering dietary linoleic acid intake, the circulating levels of 13-HODE significantly decreased in the plasma of the participants. Furthermore, our study showed a transfer of hexanal from the apical to the basolateral side of HGT-1, which is in accordance with findings from previous studies showing, first, an incorporation of hexanal by gastric rat tissue after administration of linoleic acid hydroperoxides during four hours, and, second, even an accumulation thereof in the rats’ livers after intragastric administration of a radioactive labelled mixture of aldehydes 15 h post load [10,28]. Here we presented results indicating that the oxidation products of linoleic acid and their decomposition products might be absorbed by human gastric cells, thereby making 13-HODE available for further biological effects, although quantitative studies using stable isotope labelled lipid peroxidation products subjected to mimicked gastric digestion are needed to quantitate metabolic conversions and uptake kinetics. Furthermore, 13-HODE could function as a biomarker for the assessment of the oxidative status caused by dietary intake of oxidized lipids in vivo and in early detection of diseases, which was demonstrated in one study showing elevated levels of 9-/13-HODE in patients with nonalcoholic steatohepatitis compared to patients with steatosis [29,30]. Although hexanal has been shown to be a potential biomarker for some cancer types, such as lung or breast cancer [31,32], it also could be used as a marker of oxidized dietary fat intake.

In addition to our results indicating absorption of linoleic acid oxidation products by gastric cells, we could show that linoleic acid peroxidation products, 13-HpODE and hexanal, as opposed to unoxidized linoleic acid, influenced parameters related to amino acid and protein biosynthesis after a six-hour incubation in human gastric cells on genomic and metabolic levels. Genomic RNA regulation showed that a treatment with linoleic acid, 13-HpODE, and hexanal in tested concentrations affected genes, among others, which have been shown to be involved in transcription and translation [33]. However, a regulation on the genomic level does not necessarily result in an expression on the metabolic level. Moreover, metabolomic analysis showed that an incubation with linoleic acid had more pronounced effects on glycerophospholipid metabolism.

The integrated pathway analysis combining genomic and metabolomic results of linoleic acid and its peroxidation products, 13-HpODE and hexanal, demonstrated a significant impact of the linoleic acid peroxidation products on pathways related to amino acids in this pre-absorptive cell model. However, combining the effects of linoleic acid on genomic and metabolic levels eliminates the impact of linoleic acid on the regulation of amino acid metabolism. Instead, it could be shown that 13-HpODE and hexanal increased the concentrations of valine, leucine, and isoleucine, which are branched-chain amino acids. Branched-chain amino acids are associated with a broad spectrum of physiological effects in vivo, such as protein synthesis [34]. It is suggested that branched-chain amino acids, e.g., leucine, act as signaling molecules regulating protein synthesis by influencing mRNA translation, thus enhancing protein synthesis [35]. Zhang et al. [36] could also demonstrate that a thermally processed diet significantly affected amino acid profiles of fish. In the current study, the coincident rise of the branched chain amino acids after incubation of HGT-1 cells with 100 µM peroxidized lipids for six hours suggests a regulation of the branched-chain amino acid metabolism. Literature evidence has suggested elevated leucine levels to decrease lipogenic enzymes, fatty acid synthase and acetyl-coenzyme A carboxylase and corresponding upstream proteins [37]. Thus, leucine has been suggested to improve lipid metabolism and metabolic disorders [38], known to be associated with gastric cancer. Therefore, peroxidation of linoleic acid might be crucial in influencing cellular amino acid metabolism, thereby affecting metabolic health.

## 4. Materials and Methods 

### 4.1. Chemicals

13-Hydroperoxy-9*Z*,11*E*-octadecadienoic acid (13-HpODE, purity: 95.1%) and (^13^C_18_)-13-hydroperoxy-9*Z*,11*E*-octadecadienoic acid ([^13^C_18_]-13-HpODE, purity: 92.0%) were synthesized as described in a previous publication of our group [39]. (±)-13-Hydroxy-9*Z*,11*E*-octadecadienoic acid (13-HODE, purity: ≥98%) was bought from Cayman Chemical (Cayman Europe, Tallinn, Estonia). Hexanal-d_12_ (98.5 atom% D, purity: 96%) was purchased from C/D/N Isotopes Inc. (Quebec, Canada). All other chemicals were purchased from Sigma-Aldrich (Vienna, Austria) unless otherwise stated.

### 4.2. Cell Culture

The human gastric adenocarcinoma (HGT-1) cell line was first characterized in 1982 [40]. Although HGT-1 cells do not secrete mucus, this parietal cell line does express all functional genes needed for proton secretion as key mechanism of gastric secretion. The applicability of the HGT-1 cell model to evaluate proton secretion was confirmed in an in vivo study [41]. Specifically, formation of intracellular cAMP by histamine is a common feature of HGT-1 cells and parietal cells in healthy gastric mucosa. We therefore consider the characteristics of HGT-1 cells being comparable with the physiology of parietal cells in the human stomach. HGT-1 cells were obtained as gift from Dr. C. Laboisse (Laboratory of Pathological Anatomy, Nantes, France) and cultured in DMEM with 4 g/L glucose supplemented with 10% fetal bovine serum, 2% L-glutamine and 1% penicillin/streptomycin under standardized conditions (37 °C, 5% CO_2_ and 95% humidity) until reaching confluency. Effects of the tested substances on the cells’ viability at a concentration of 100 µM revealed maximum exposure times of 30 min for hexanal and 6 h for linoleic acid and 13-HpODE not showing statistically different effects from medium-only treated cells by means of an MTT assay (data not shown). The MTT assay determines the reduction of 3-[4,5-dimethylthiazole-2-yl]-2,5-diphenyltetrazolium bromide (MTT) to the MTT-formazan which is catalyzed by mitochondrial succinate dehydrogenase.

### 4.3. Exposure Study

After reaching about 80% confluency, the HGT-1 cells were seeded onto Snapwell inserts with a polycarbonate membrane (tissue culture treated, 12 mm diameter, 0.4 μm pore size) (Costar, Corning Inc., New York, NY, USA) using DMEM supplemented with 20% fetal bovine serum (FBS) with a density of 1 × 10^5^ cells/cm^2^. On day one post-seeding, 250 µL of DMEM supplemented with 20% FBS were added to each apical compartment of the Snapwell inserts. On day three post seeding, a complete medium change (DMEM + 20% FBS) was performed in the apical and basolateral compartment, whereas the experiment was conducted on Day 5. The integrity of the cells’ monolayer was monitored every day by measuring the transepithelial electrical resistance (TEER) by means of a volt/ohm meter (EVOM-24, World Precision Instruments Inc., Florida, FL, USA). The exposure experiment was done on Day 5 since there was a plateau of the TEER reached after four to eight days post seeding and additionally a phenol red permeability assay, performed on the experiment day, showed a penetration of phenol red of about 8% from the apical to the basolateral compartment after one hour without changing the TEER, confirming the integrity of the cell monolayer [42].

On the day of the exposure experiment, after measuring TEER and phenol red permeability, the supernatant of the basolateral and apical compartment was aspirated, and cells were washed twice with phosphate-buffered saline (PBS). Afterwards, 500 µL of incubation solution (100 µM linoleic acid, 100 µM 13-HpODE or 100 µM hexanal in DMEM, respectively) were added to the apical compartment of the Snapwell insert, whereas 3 mL of medium (DMEM without FBS) was added to the basolateral compartment prior to sealing of the Snapwell plate. These concentrations for incubations were chosen for further assays because they represent oil-characteristic amounts of the test substances [39,43,44,45]. After an incubation of linoleic acid and 13-HpODE for six hours and an exposure to hexanal for 0.5 h (37 °C, 5% CO_2_, 95% humidity), which mimics relevant gastric retention time of food lipids, the supernatants from the basolateral and apical compartment were collected. Afterwards, pyrogallol (2 mM) was added to each sample and covered with argon, to avoid further oxidation, the samples were stored at −80 °C for further experiments.

After collection of the samples, the cells were lyzed. For that, the membrane of the apical compartment was washed with 500 µL PBS and 200 µM trypsin were added. After five minutes of trypsination (37 °C, 5% CO_2_, 95% humidity), 200 µL medium were added and mixed well. After transferring the samples into reaction tubes, the Snapwell membrane was rinsed thrice with 400 µL H_2_O (double-distilled), which was collected in the same reaction tube. Cell lysis was performed by means of three consecutive freeze-thaw cycles (30 s in liquid nitrogen then 3 min in heat block at 90 °C). Afterwards, centrifugation was done at 16,100× *g* at 4 °C for 10 min, the supernatants were collected, mixed with pyrogallol [2 mM] and covered with argon. The samples were stored at −80 °C for further experiments.

### 4.4. Quantitation of Linoleic Acid Peroxidation Products

The samples, obtained from the exposure assay after linoleic acid and 13-HpODE incubation, were spiked with ^13^C_18_-13-HpODE, filtered through a nylon filter (0.2 µm, Phenomenex, Aschaffenburg, Germany) and subjected to quantitative analysis of 13-HpODE and 13-HODE according to Pignitter et al. [39] with slight modifications. Briefly, the samples were analyzed with a quadrupole liquid chromatography-mass spectrometry system (LCMS-8040, Shimadzu, Vienna, Austria) using a C18 column (Kinetex EVO, 150 mm × 4.6 mm; 5 µm diameter, Phenomenex, Aschaffenburg, Germany). The mobile phase was composed of 0.1% formic acid in methanol and double-distilled water. A flow rate of 1.0 mL/min was used, and the gradient elution program started with 60% methanol/40% double-distilled water, reaching a plateau of 90% methanol/10% double-distilled water after 10 min and returned to starting conditions after 30 min. The following MS settings were used for recording multiple reaction mode (MRM(-)): nebulizing gas flow, 3 L/min; drying gas flow, 10 L/min; desolvation line temperature, 250 °C; heat block temperature, 150 °C; CID gas, argon; collision energy, 15 V; MRM (precursor ion *m*/*z* -> product ion *m*/*z*), 13-HpODE (311 -> 113), [^13^C_18_]-13-HpODE (329 -> 120), 13-HODE (295 -> 277). Quantitation of 13-HpODE was done in MRM(-) mode using stable isotope dilution assay selecting transition ions *m*/*z* 113 for 13-HpODE and *m*/*z* 120 for ^13^C_18_-13-HpODE, as internal standard [46]. 13-HODE was quantitated with external calibration using the transition ion *m*/*z* 277 [47]. Limit of detection (LOD = 0.1 µg/mL) was determined by a signal-to-noise ratio of 3, while the limit of quantitation (LOQ) was calculated based on a signal-to-noise ratio of 9.

### 4.5. Quantitation of Hexanal

After adding hexanal-d_12_ as an internal standard, the sample, obtained from the exposure assay, was extracted with 2 × 500 µL hexane, filtered through a nylon filter (0.2 µm, Phenomenex, Aschaffenburg, Germany) and subjected to quantitative analysis as reported previously, with minor modifications [21]. In short, the sample was injected using splitless mode and analysis was performed using a GC-MS (GCMS -QP 2010 Ultra, Shimadzu, Vienna, Austria) with a capillary column (ZB-WAX Zebron, 30 m × 0.25 mm i.d., 0.25 μm film thickness) for separation. Measurement parameters are shown in greater detail by Pignitter et al. [21]. Stable isotope dilution analysis was applied as quantitation method selecting fragment ions *m*/*z* 72 for hexanal and *m*/*z* 80 for hexanal-d_12_ in SIM mode. Limit of detection (LOD = 0.02 µg/mL) was determined by a signal-to-noise ratio of 3, while the limit of quantitation was calculated based on a signal-to-noise ratio of 9.

### 4.6. Genome Analysis of RNA Regulation

Effects of linoleic acid, 13-HpODE and hexanal on genomic level in HGT-1 cells were examined using a customized cDNA microarray as described before [48]. Briefly, after cells were seeded in a density of 10 × 10^5^ cells/cm^2^, on the next day cells were incubated with 100 µM linoleic acid, 100 µM 13-HpODE or 100 µM hexanal in DMEM (+ 1%FBS) for six hours at standardized conditions (37 °C, 5% CO_2_, 95% humidity), respectively, and subsequently RNA was isolated using the RNeasy Mini Kit (Qiagen, Hilden, Germany). cDNA reverse transcription was carried out with Cy3-labeled primers (Tebu Bio, Offenbach, Germany) according to Ouellet et al. [49] After hybridization at 42 °C for 20 h, the microarrays were scanned with an Axon GenePix 4400 A microarray scanner (Molecular Devices, Sunnyvale, CA, USA). Fluorescence intensities were analyzed using NimbleScan 2.1 software and for normalization, the background intensities were corrected using robust multichip analysis (RMA). Moreover, four non-regulated reference genes (PPIA, GAPDH, TBP, UBC) were used for normalization of the fluorescence intensities of each microarray chip.

### 4.7. Metabolomic Analysis

To examine whether linoleic acid, 13-HpODE, and hexanal have an effect on metabolomic level in HGT-1 cells, targeted metabolomic analysis was performed using the AbsoluteIDQ p150 Kit (BIOCRATES Life Sciences AG, Innsbruck, Austria). Cells were seeded in a density of 6 × 10^5^ cells/cm^2^ and cultivated for one day. Afterwards, cells were washed with 500 µL PBS and incubated with 100 µM linoleic acid, 100 µM 13-HpODE, or 100 µM hexanal in DMEM (+ 1% FBS) for 6 h (37 °C, 5% CO_2_, 95% humidity), respectively. After incubation, cells were washed twice with 500 µL ice-cold PBS and 300 µL H_2_O (double-distilled) were added. Cell lysis was carried out by means of three consecutive freeze-thaw cycles (30 s in liquid nitrogen <-> 3 min in heat block at 90 °C). After collection of the cell lysates with a cell scraper and rinsing the well with 200 µL H_2_O (double-distilled), cells were centrifuged at 20,000× *g* for 10 min at 4 °C. Finally, the supernatant was removed, covered with argon and stored at −80 °C until analysis.

Quantitative analysis was done according to the manufacturer’s manual, which allows the determination of 163 endogenous metabolites from 5 compound classes (amino acids, acylcarnitines, sphingolipids, phospholipids, and the sum of hexoses). Analysis was carried out with a HPLC using electrospray-flow injection analysis followed by tandem mass spectrometry and isotope-labeled internal standards were used for quantitation. For validation of the kit and calculation of the metabolites’ concentrations (µM) the MetIDQ Boron 5.4.8. software (BIOCRATES Life Sciences AG, Innsbruck, Austria) was used [50].

### 4.8. Statistical Analysis

Analyses were carried out with three to four independent biological replicates and one to two technical replicates. Statistical analyses were performed using SigmaPlot 11 (Systat Software Inc., Chicago, IL, USA). Two-way ANOVA followed by the Holm–Sidak post hoc test was used for determining statistical differences. *P*-values below 0.05 indicate statistical significance. Data are shown as mean ± standard deviation (SD) unless stated otherwise.

## 5. Conclusions

Stored and heat-treated oils are considerable sources for dietary lipid peroxidation products. There is evidence that dietary primary and secondary peroxidation products of linoleic acid, the most abundant polyunsaturated fatty acid in vegetable oils, are degraded in the stomach before being absorbed into the body. The research here presented results confirming this hypothesis, strongly suggesting that linoleic acid and its primary peroxidation product, 13-HpODE, were degraded into the corresponding hydroxy fatty acid, 13-HODE, which is very likely absorbed by gastric cells. However, it has to be noted that the degradation pathway(s) of 13-HpODE leading to the formation of 13-HODE were not investigated but need to be unraveled in future studies by means of, e.g., the CAMOLA technique [51]. For hexanal, a secondary peroxidation product of linoleic acid, results also indicated absorption by the gastric cells. Moreover, an integrated pathway analysis revealed that the pathways related to amino acid biosynthesis were mainly affected by linoleic acid primary and secondary peroxidation products in gastric cells. Elevated levels of branched-chain amino acids are supposed to improve lipid metabolism, potentially indicating beneficial effects of peroxidized lipids on metabolic health. To fully elucidate and verify effects of individual primary and secondary linoleic acid peroxidation products on cellular pathways related to amino acid metabolism, further in vivo studies are warranted.

## Figures and Tables

**Table 1:** Quantitation of 13-HODE and hexanal in different compartments (apical, lysate, basolateral) of HGT-1 cells after six-hours incubation with 100 µM linoleic acid and 100 µM 13-HpODE or 0.5 h incubation with 100 µM hexanal ^a^.

Incubation Substance	Linoleic Acid	13-HpODE	Hexanal
Quantitation of	13-HODE [µM]	13-HODE [µM]	Hexanal [µM]
**apical**	1.11 ± 0.05 ^a^	1.22 ± 0.05 ^a^	3.15 ± 0.62 ^b^
**lysate**	n.d.	n.d.	n.d.
**basolateral**	1.12 ± 0.05 ^a^	2.09 ± 0.53 ^b,^*	0.20 ± 0.13 ^c,^*

^a^ Data are displayed as mean ± SD (*n* = 3–4, tr = 1–2). Statistically significant differences were analyzed using two-way ANOVA (*p* ≤ 0.01), followed by the Holm–Sidak post hoc test (*p* ≤ 0.05). ^a,b,c^ Different letters in a row indicate significant differences between the three treatments (*p* ≤ 0.05). Asterisks (*) indicate significant differences within one treatment between apical and basolateral compartments (*p* ≤ 0.05).

**Table 2:** Significantly enriched pathways (*p* < 0.05) based on the integrated methods pathway analysis after six-hours incubation with 100 µM linoleic acid, 100 µM 13-HpODE, or 100 µM hexanal in HGT-1 cells ^a^.

Compound	Pathway	Hits (Official Gene Symbol, KEGG Compound Entry)	*p* Value	Topology
Linoleic acid	Pyrimidine metabolism	UMPS, POLR1A, CANT1, NTSC3, TXNRD2, TYMP, TYMS	0.036	0.37
	Cyanoamino metabolism	5HMT1, C00037	0.040	0.75
13-HpODE	Aminoacyl-tRNA biosynthesis	C00123, C00037, RARS2, C00148, FARSB, C00073	< 0.001	0.12
	Arginine and proline metabolism	GLS2, ASS1, C00148, GOT2, ALDH3A2	0.004	0.17
	Linoleic acid metabolism	C00157, PLA2G6, CYP2C8	0.006	0.88
Hexanal	Valine, leucine and isoleucine biosynthesis	PDHA1, C00183, C00123	< 0.001	0.45
	Aminoacyl-tRNA biosynthesis	C00183, C00123, C00037, C00062, C00073	< 0.001	0.07
	Pantothenate and CoA biosynthesis	VNN2, C00183	0.030	0.13
	Arginine and proline metabolism	NOS2, C00062, C00077	0.044	0.27

^a^*p*-values less than 0.05 indicate a statistical significance compared to non-treated control cells.

## Supplementary Materials

The following are available online at https://www.mdpi.com/1420-3049/24/22/4111/s1, Table S1: Annotation clusters from microarray probes, Table S2: Metabolic pathway analysis.
