# Supplementary material for: Exposure of Human Gastric Cells to Oxidized Lipids Stimulates Pathways of Amino Acid Biosynthesis on a Genomic and Metabolomic Level
Source: Molecules. 2019 Nov 14;24(22):4111. doi: 10.3390/molecules24224111 (PMC6891525; doi:10.3390/molecules24224111)
Supplement: Supplementary file 1 [file molecules-24-04111-s001.pdf]

Supplemental tables

Table S1. Selected annotation clusters with enrichment scores ≥ 1.3 found by DAVID using input data of microarray probes with fold-changes below 0.8 or above 1.2 after six-hours incubation with 100 µM linoleic acid, 100 µM 13-HpODE or 100 µM hexanal in HGT-1 cells.

Compound	Annotation Cluster	Enrichment Score		Count	P value	Benjamini
Linoleic acid	UP_KEYWORDS	Spliceosome	1.33	13	0.011	0.43
	UP_KEYWORDS	mRNA processing		25	0.014	0.43
	UP_KEYWORDS	mRNA splicing		19	0.043	0.63
13-HpODE	UP_SEQ_FEATURE	region of interest: Modulating	1.62	4	0.0012	0.70
	UP_SEQ_FEATURE	DNA-binding region: Nuclear receptor		5	0.0053	0.66
	UP_SEQ_FEATURE	zinc finger region: NR C4-type		5	0.0053	0.66
	INTERPRO	Zinc finger, nuclear hormone receptor-type		5	0.0058	0.61
	INTERPRO	Steroid hormone receptor		5	0.0063	0.56
	SMART	ZnF_C4		5	0.0067	0.39
	INTERPRO	Nuclear hormone receptor, ligand-binding, core		5	0.0068	0.52
	SMART	HOLI		5	0.0078	0.32
	UP_SEQ_FEATURE	region of interest: Hinge		4	0.0093	0.74
	UP_SEQ_FEATURE	region of interest: Ligand-binding		4	0.011	0.72
	INTERPRO	Zinc finger, NHR/GATA-type		5	0.013	0.71
	GOTERM_MF_DIRECT	steroid hormone receptor activity		5	0.014	0.44
	GOTERM_MF_DIRECT	transcription initiation from RNA polymerase II promoter		6	0.10	0.93
Hexanal	KEGG_PATHWAY	Neuroactive ligand-receptor interaction	3.28	17	0.00000000088	0.00000012
	UP_SEQ_FEATURE	lipid moiety-binding region: S-palmitoyl cysteine		11	0.00000098	0.0005
	UP_KEYWORDS	Palmitate		12	0.000018	0.0021
	SMART	SM01381		8	0.000035	0.003
	UP_KEYWORDS	Lipoprotein		18	0.000098	0.0077
	GOTERM_BP_DIRECT	phospholipase C-activating G-protein		6	0.00013	0.11

Table S2. Significantly enriched pathways ($p < 0.05$) based on the metabolic pathway analysis after a six-hours incubation period with 100 µM linoleic acid, 100 µM 13-HpODE or 100 µM hexanal in HGT-1 cells.

Compound	Pathway	Hits (KEGG compound entry)	P value
Linoleic acid	Glycerophospholipid metabolism	C04230	0.007
	Aminoacyl-tRNA biosynthesis	C00037, C00123	0.020
	Linoleic acid metabolism	C00157	0.049
13-HpODE	Aminoacyl-tRNA biosynthesis	C00037, C00073, C00123, C00148	<0.001
	Glycerophospholipid metabolism	C00157, C04230	0.011
Hexanal	Aminoacyl-tRNA biosynthesis	C00062, C00037, C00073, C00183, C00123	<0.001
	D-Arginine and D- Ornithine metabolism	C00062, C00077	<0.001
	Valine, Leucine, Iso- biosynthesis	C00123, C00183	0.008

p values less than 0.05 indicate statistical significance vs. non-treated control cells (n=3-4).
